# Job Crafting in Nursing: Mediation between Work Engagement and Job Performance in a Multisample Study

**DOI:** 10.3390/ijerph191912711

**Published:** 2022-10-05

**Authors:** Gabriela Topa, Mercedes Aranda-Carmena

**Affiliations:** 1 Faculty of Psychology, National Distance Education University (UNED), 28040 Madrid, Spain; 2 Universidad Autónoma de Chile, Santiago 7500912, Chile; 3 Department of Psychology, Faculty of Health Sciences, Universidad Rey Juan Carlos, 28933 Madrid, Spain

**Keywords:** job crafting, nursing, work engagement, job performance, collaborative crafting, cognitive crafting, task-related crafting, relational crafting

## Abstract

Job crafting is considered a specific form of proactive behavior whereby workers actively change the actual or perceived characteristics of their jobs in order to better match the demands placed on them and the resources available. As nursing could be considered a stressful profession, job crafting is proposed as a mediator between nurses’ work engagement and job performance. Hence, the main objective of this study was to provide empirical evidence on job crafting in nursing, including the three most prominent conceptualizations of the construct. The present research covers three independent empirical studies of registered or practical nurses of Spanish public and private hospitals: Study 1 (*N* = 699), Study 2 (*N* = 498), and Study 3 (*N* = 308). (3) Our results support the hypothesis that nurses’ job engagement and job-crafting behaviors can affect their job performance. Our finding corroborates that engaged nurses can act to proactively change their jobs, but comparing different job-crafting conceptualizations and measures, the current findings support that effectiveness of diverse job crafting behaviors could vary. To sum up, as the JDR approach proposed, the present study supports the position that work engagement influences job performance, as well as the mediating role of job crafting in this relationship. The current study takes this knowledge one step further by revealing that not all types of job-crafting behaviors are equally efficient and not all types are adequate for specific working environments, such as nursing.

## 1. Introduction

Nursing could be considered one of the most stressful professions, as nurses are confronted with injury, pain, and death on a daily basis [1]. The availability of resources to perform the job, the demands of patients and families, and the interaction with colleagues and the constantly changing work environment all contribute to nursing stress [2,3]. In this complex work environment, job crafting is considered a specific form of proactive behavior whereby workers actively change the actual or perceived characteristics of their jobs in order to better match the demands placed on them and the resources available. Although the first empirical studies were conducted in other professions, the relationships between job crafting, work engagement, and job performance in nursing are accumulating increasing empirical evidence [4,5,6]. Job crafting is a complex construct that has given rise to various conceptualizations, but most of the empirical studies in nursing use the measures proposed by Tims and Bakker [7] and Tims et al. [8].

The main objective of this study was to provide empirical evidence about job crafting in nursing, including the three most prominent conceptualizations: job crafting as the increase of structural resources, social resources, and challenging demands in tandem with the decrease of hindering task requirements [7,8], as well as the alternative conceptualizations of job crafting as a behavior that is executed individually or collectively [9,10] and job crafting as a behavior that can take three forms: task crafting, relational crafting, and cognitive crafting [11,12]. This broader evidence provides stronger empirical support for the usefulness of job crafting in nursing. At the same time, the findings allow us to compare the different conceptualizations with each other in order to learn which dimensions of job-crafting behavior are most relevant in the health-related working environment.

### 1.1. Work Engagement and Job Performance: Mediating Role of Job Crafting

As job demands–resources (JDR) theory points out, demands are understood as the physical, psychological, organizational, or social aspects that require efforts from the worker, while resources are considered those aspects of the job that reduce demands, facilitate the achievement of objectives, or stimulate personal development. Demands and resources trigger two parallel processes (burnout and engagement) that ultimately affect job performance. Work engagement has been described as a satisfying psychological state characterized by vigor, dedication, and absorption in work [13].

Empirical studies and meta-analytic reviews repeatedly support the positive relationship between work engagement and performance, for example, through the inclination to exhibit extra-role behaviors or to be involved in organizational goals [14]. Much recent research on work engagement in nursing has firmly established the relationship with job performance in this specific work context. Specifically, empirical studies on Spanish nursing professionals showed that work engagement impacts workers’ occupational health and patients’ care and satisfaction [15,16]. Most of the studies have deeply explored the personal and psychosocial antecedents of work engagement among nurses. For instance, Pérez-Fuentes and her colleagues [17] showed that higher levels of emotional intelligence predicted engagement, while Martos-Martínez et al. [18] established that four of the “Big Five” personality factors affect work engagement through the mediation of positive affect and cognitive empathy. Studies from other cultures have provided evidence on the antecedent roles of spiritual leadership [19] and work motivation on the maintenance of engagement [20], while some studies recently showed the impact of COVID-19 pandemic-related stress on work engagement [21]. Despite the abundance of recent developments in work engagement among nurses, its impact on job performance should be more firmly established. Hence, we explored this relationship using different Spanish nursing samples in order to obtain wider evidence on the stability of this relationship across diverse organizations. Therefore, in the present research, work engagement was expected to directly and positively predict job performance (Hypothesis 1).

As Tims, Bakker, and Derks posited [22], job crafting may arise as a response from engaged workers seeking to increase their resources or from burned-out workers seeking to reduce their demands. Starting from a key premise of COR theory (conservation of resources), according to which resources “travel in caravans,” the abundance of energy resources is more likely to mobilize and generate profits from new resources. Therefore, in the present research, job crafting is predicted to mediate the relationship between work engagement and job performance.

### 1.2. Job Crafting: Diverse Conceptualizations and Measures

The most widespread conceptualization of job crafting is based on the RPG approach. Tims and Bakker [7] proposed that employees initiate this behavior to change their real or perceived job demands and resources. These proactive employee behaviors are intended to: (a) increase their structural resources, (b) increase their social resources, (c) reduce disruptive demands, or (d) increase challenging demands. The most widespread conceptualization of job crafting is based on the RPG approach. Tims and Bakker [7] proposed that employees initiate this behavior to change their real or perceived job demands and resources. These proactive employee behaviors are intended to: (a) increase their structural resources, (b) increase their social resources, (c) reduce disruptive demands, or (d) increase challenging demands. As a consequence, most empirical studies in nursing and the health professions in general have used this conceptualization and the corresponding measure [23].

Despite the popularity of Tims and her colleagues’ approach [22], other conceptualizations of job crafting exist. Leana, Appelbaum and Shevchuk [9] criticized the previous approach because it considered job crafting an exclusively individual activity. These authors pointed out that the effects of collaborative job crafting were more powerful than the effects of individual job crafting. Workers participate in similar work processes, relate to each other, and live common experiences. Therefore, “they can jointly determine how to modify the work to meet their shared goals” [9]. According to this research, job crafting is made up of two constructs: individual crafting and collaborative crafting. Cheng, Chen, Teng and Yen [24] explained that through individual crafting, employees actively alter the boundaries of their tasks, while in collaborative crafting, employees work together to review the work process. Leana et al. [9] and other studies [25,26] provided empirical results on the relationships between job crafting and organizational outcomes. While collaborative crafting was related to higher quality of care, job satisfaction, and organizational commitment, individual crafting did not predict such outcomes. Chen, Yen and Tsai [27] found that both types, individual and collaborative crafting, had close relationships with work engagement. More recently, Llorente and Topa [28] found positive relationships between individual and collaborative job crafting with personal indicators of well-being among Spanish employees.

Wrzesniewski and Dutton [11] anticipated that job crafting could take three forms: task crafting, relational crafting, or cognitive crafting. Task crafting refers to changes made by individuals regarding type or number of activities, for instance, including new tasks so their job achieves a better fit to their personality. The crafting of relational aspects of the job includes changes related to interactions with others, such as introducing more contact with the recipients of their work. Cognitive crafting reflects the alterations in the way the individual perceives their job, for example, seeing their job as part of a larger organizational effort to contribute to the welfare of clients. Consistent with this conceptualization, the Job Crafting Questionnaire was developed by Slemp and Vella-Brodrick [12], including task, relational, and cognitive crafting, and recently adapted to non-English-speaking populations, such as Germans [29] and Spanish [26].

Although scarcer, empirical research using the Job Crafting Questionnaire has also shown relationships between job crafting and positive consequences: a study on the relationships between individual outcomes and job crafting showed that task, relational, and cognitive job crafting were related significantly connected with need satisfaction, which in turn is related to subjective and psychological well-being [30], and job crafting showed positive relationships with perceived autonomy support, job satisfaction, and work-related positive affect, as well a negative relationship with work-related negative affect [31].

Despite the fact that wider evidence on the positive impact of job crafting on personal and organizational outcomes has been provided by different studies, specific findings among nurses are relatively scarce. Moreover, due to the fact that detailed working procedures are used in medical environments, with the main objective of reducing errors that negatively affect patients’ care, job crafting among nurses is still a topic under debate [16]. Hence, the present article is devoted to providing firm evidence on the usefulness of job crafting in nursing activities based on more than one sample. We predicted that some differences would be found in the mediating role of different dimensions of job crafting and the relationship between work engagement and job performance (Hypothesis 2). Since there is no consistent prior evidence, the direction of the differences cannot be hypothesized.

## 2. Materials and Methods

### 2.1. Design and Participants

The present research included three independent empirical studies conducted between 2021 and 2022: Study 1 (*N* = 699), Study 2 (*N* = 498), and Study 3 (*N* = 308). Demographic characteristics of the three samples are shown in Table 1.

### 2.2. Instruments

The same variables were assessed in the three independent studies: work engagement and job performance using the same instruments. Information about the psychometric properties of the scales used is provided in the diagonal of tables. Regarding the assessment of job crafting, in order to test hypothesis 2, three different scales were used, as explained below.

#### 2.2.1. Work Engagement

Work engagement was assessed with the nine-item version of the Utrecht Work Engagement Scale (UWES) [32]. Example items are: “At my job I feel strong and vigorous,” “I am enthusiastic about my job,” and “I am immersed in my work.” All items were scored on a five-point rating scale ranging from 1 (never) to 5 (always). One overall score for work engagement was created.

#### 2.2.2. Job Performance

In the present research, job performance was assessed with the Performance Scale (In-Role Behaviors) developed by Williams and Anderson [33], consisting in 7 items. Participants were asked to assess what items adequately described the way they performed their work, e.g., “You adequately complete assigned duties,” “You meet formal performance requirements on the job,” or “You neglect aspects of the job you are obliged to perform” (reversed item). Participants could respond to these items on a 5-point Likert-type scale (1 = strongly disagree to 5 = strongly agree).

#### 2.2.3. Job Crafting

For Study 1, job crafting was measured with the four subscales from the Job Crafting Scale [8] that are indicative of increasing structural job resources and social job resources, as well as increasing challenging job demands, such as proactive involvement in new projects and decreasing hindering job demands, for example, by decreasing the number of emotional interactions or cognitive tasks. Example items are “I ask others for feedback on my job performance,” “I try to develop my capabilities,” and “I ask colleagues for advice.” All items were scored on a five-point rating scale ranging from 1 (never) to 5 (always).

For Study 2, the Spanish version of the Individual and Collaborative Crafting Scale [9] validated by Llorente and Topa [28]. The scale is composed of two subscales that assess the dimensions of individual and collaborative crafting, with 6 items of each. Examples of individual crafting items are: “You propose, on your own, new approaches to improve your work” and “You change, on your own, work procedures that you believe are not productive.” Likewise, examples of collaborative crafting are: “You work together with your colleagues to come up with new approaches to improve your work” and “You decide together with your colleagues to bring other materials for the job.” The Likert-type response scale ranged from 1 (strongly disagree) to 5 (strongly agree).

For Study 3, the Spanish version [26] of the Job Crafting Questionnaire [12] was used. The JCQ consists of 19 items distributed in three subscales (task—seven items, cognitive—five items, and relational—seven items), and they are answered on a 5-point Likert-type scale (1 = strongly disagree to 5 = strongly agree).

Demographic characteristics (hospital tenure, professional category, age, gender, and educational level) were answered by participants at the end of the survey. In Study 2, data were also collected on the type of hospital (private vs. public) and the region of Spain where the hospital was located.

### 2.3. Procedure

Data for the three studies were collected from Spanish-registered practical nurses. Participants were recruited with the collaboration of other nurses enrolled as PhD and master’s students during the 2021 and 2022 courses (being the first author his/her advisor), who received in exchange academic credits, following the procedure suggested by Demerouti and Rispens [34] for student-recruited samples. Potential participants were informed about the research objectives, anonymity, voluntariness, and the possibility of leaving the study at any time, and the research group did not know any personal data of the participants. All the participants had initially signed an informed consent and then completed the survey using a Google Forms questionnaire. The National Distance Education University Bio-Ethical Committee approved this research.

### 2.4. Statistical Analyses

To determine bivariate correlations, Pearson’s correlation coefficient analyses were conducted. The hypotheses were tested using the Process macro for SPSS [35]. To be specific, the direct effect of work engagement (X, the independent variable) on job performance (Y, the criterion variable) was tested using Model 4 (Hypothesis 1), as well as the mediating effect of job-crafting dimensions (M1, M2, etc., the mediating variables) in the relationship between work engagement and job performance (Hypothesis 2). These models were tested using 5000 bootstrap samples in order to obtain reliable estimates of standard errors and confidence intervals. When zero is not included in the 95% bias-corrected confidence interval, it may be concluded that the parameter is significantly different from zero at *p* < 0.05. Age and hospital tenure were included as covariates, given their association with work engagement and job performance. Analyses were performed using SPSS 25.0 (SPSS Inc, Chicago, IL, U.S.A.).

## 3. Results

### 3.1. Descriptive Results

#### Bivariate Correlations

For Study 1, the Pearson’s correlation matrix is displayed in Table 2. As displayed on the diagonal of this table, all scales have satisfactory reliabilities, with Cronbach’s alpha coefficients higher than 0.70. Relationships between age and work engagement were nonsignificant, as well as between age and job performance.

For Study 2, the Pearson’s correlation matrix is displayed in Table 3 (*N* = 498). As displayed on the diagonal of this table, all scales have satisfactory reliability, with Cronbach’s alpha coefficients higher than 0.70.

For Study 3, the Pearson’s correlation matrix is displayed in Table 4 (*N* = 308). As displayed on the diagonal of this table, all scales have satisfactory reliabilities, with Cronbach’s alpha coefficients higher than 0.70.

### 3.2. Mediation Analyses

All the analyses were aimed at assessing the indirect effect of work engagement on job performance through job-crafting dimensions. For Study 1, the overall model was significant (F (7.662) = 12.421, *p* < 0.001, *R*^2^ = 0.116). Results indicated a positive total effect of work engagement on job performance (B = 0.111, SE = 0.023, 95% CI [0.065; 0.157], *p* = 0.000) and a nonsignificant negative direct effect of work engagement on job performance (B = −0.011, SE = 0.029, 95% CI [−0.068; 0.045], *p* = 0.696), failing to support Hypothesis 1. Regarding Hypothesis 2, the four indirect effects of job-crafting dimensions between these variables, only the increase of structural resources showed a significant indirect effect (B = 0.103, SE = 0.014, 95% CI [0.077; 0.133]). Subsequent Sobel tests supported this result (z = 6.123, *p* = 0.000). Comparison among the specific indirect effects showed that only C1, C2, and C3 were significant in involving increase of structural resources minus the other three dimensions of job crafting. In our study, age and hospital tenure were not significantly related to job performance. Taken together, these results indicated a significant mediating effect of job-crafting behaviors increasing structural resources in the relationship between work engagement and job performance.

For Study 2, the overall model was significant (F (3.477) = 33.066, *p* < 0.001, *R*^2^ = 0.19). Results indicated a positive total effect of work engagement on job performance (B = 0.882, SE = 0.091, 95% CI [0.704; 1.061], *p* = 0.000) and a significant positive direct effect of work engagement on job performance (B = 0.760, SE = 0.101, 95% CI [0.560; 0.960], *p* = 0.000), fully supporting Hypothesis 1. Regarding Hypothesis 2, the two indirect effects of job-crafting dimensions between these variables, only the individual crafting behavior showed a significant indirect effect (B = 0.118, SE = 0.048, 95% CI [0.026; 0.219]). Subsequent Sobel tests supported this result (z = 2.3903, *p* = 0.016). Comparison of the specific indirect effects showed that C1, involving individual crafting behaviors minus collaborative job crafting, was not significant. In our study, age and hospital tenure were not significantly related to job performance. Taken together, these results indicated a significant mediating effect of individual job-crafting behaviors in the relationship between work engagement and job performance.

For Study 3, the overall model was significant (F (6.286) = 2.795, *p* < 0.05, *R*^2^ = 0.06). Results indicated a positive total effect of work engagement on job performance (B = 0.104, SE = 0.034, 95% CI [0.035; 0.173], *p* = 0.003) and a nonsignificant positive direct effect of work engagement on job performance (B = 0.037, SE = 0.047, 95% CI [−0.056; 0.131], *p* = 0.000), failing to support Hypothesis 1. Regarding Hypothesis 2, for the three indirect effects of job-crafting dimensions between these variables, only the task-related crafting behavior showed a significant indirect effect (B = 0.049, SE = 0.022, 95% CI [0.008; 0.099]). Subsequent Sobel tests supported this result (z = 1.9268, *p* = 0.05). Comparison of the specific indirect effects showed that they were not significant. In our study, age and hospital tenure were not significantly related to job performance. Taken together, these results indicated a significant mediating effect of Task-related job crafting behaviors in the relationship between work engagement and job performance, partially supporting Hypothesis 2.

## 4. Discussion

The present study supports the hypothesis that nurses’ job engagement and job-crafting behaviors can affect their job performance. To date, a large body of empirical evidence has recognized the positive impact of work engagement on strategic work outcomes, such as employees’ in-role performance [23]. The present findings are consistent with previous research on the desirable impact of nurses’ work engagement on valued organizational outcomes in terms of job performance and personal well-being [17,18]. Our finding corroborates the central assumptions of the JD-R approach by showing that engaged nurses can act to proactively change their jobs to match demands and resources to their skills, competence, and knowledge, which in turn represents fertile ground for increased job performance. Therefore, increasing the person–job fit could facilitate in-role performance among nurses.

Furthermore, the current research suggests that a further step can be taken in this field of study. In particular, comparing different job-crafting conceptualizations and measures, the current findings support that effectiveness of diverse job-crafting behaviors could vary, and our data are consistent with previous findings [36].

In the first study, indirect effects only were exerted by the strategies aimed at increasing structural resources, while the impact of the other three dimensions remained nonsignificant. This result agrees with Rudolph et al.’s [23] meta-analysis, where increasing challenging demands and structural and social resources was proposed as a predictor of job performance. Despite this, only the increasing structural resources dimension was found to be an important determinant of self-rated performance, accounting for 66.44% of the variance in performance and 61% in contextual performance. An unexpected finding was that increasing social resources exerted a negative indirect effect on job performance. Perhaps engaged nurses that orient their crafting behaviors to the increase of their social contacts at work, instead of improving their performance, reduce it by distracting their attention from the tasks or experiencing intense emotions that could interrupt their concentration on the duties. An unexpected finding in this study was the absence of significant relationships between age and both work engagement and job performance, since this is contradictory to previous research [14]. Despite this, our finding is consistent with other previous studies, such as Pérez-Fuentes et al. [17], who showed a negative correlation between age and the three dimensions of work engagement. Perhaps our sample suffered from some kind of job insecurity and high turnover, due to the fact that age did not show a positive correlation with hospital tenure, as in the second and third studies. Nonappearance of a positive and significant relationship between nurses’ age and hospital tenure could also explain the absence of significant and positive relationships between age and engagement in this sample.

In the second study, only the individual job-crafting dimensions had significant indirect effects on job performance. This finding is consistent with the proposal of Leana et al. [9] considering both dimensions as empirically and conceptually independent concepts. Previous research has suggested a possible serial mediation of the two job-crafting dimensions. Tims et al. [2] showed that workers who operated in teams in which the members crafted their jobs together were more likely to be involved in individual crafting. According to these authors, individuals distinguished between their own behaviors and affective experiences and those of the team as a whole. Contrary to this suggestion, Llorente and Topa’s study [28] found that individual crafting was the antecedent to collaborative crafting, suggesting that individuals who craft their own jobs are more likely and more willing to collaborate in activities designed to foster team-based collaborative job crafting.

The third study showed that task-related crafting behavior was positively and significantly associated with job performance. Thus, employees that invest effort in making changes to their tasks (in order to achieve a better fit between their jobs and their personality, for instance) and alterations in the way they perceive their jobs also tend to report higher levels of job performance. Despite the fact that there are no specific hypotheses about comparisons, relationships between cognitive and relational crafting and job performance were not significant. The possibility of obtaining different results for the different dimensions of job crafting was also observed by Rudolph et al. [23], who stressed the importance of considering the different dimensions of job crafting individually, avoiding a global aggregate score. Moreover, cognitive crafting behaviors exerted a negative, but not significant effect on job performance. While task-related crafting refers to changes made by individuals regarding type or number of activities, for instance, including new tasks so their job achieves a better fit with their personality, the cognitive crafting dimension reflects the alterations in the way the individual perceives their job, e.g., seeing their job as part of a larger organizational effort to contribute to the welfare of patients. This contradictory finding suggests that not all types of crafting would be advisable for all types of jobs. In this sense, nurses’ duties entail procedures highly standardized and coordinated among several professionals involved in patients’ health care. This could be the reason that changes in the perceptions of the job would negatively impact on in-role performance.

To sum up, as the JDR approach proposed, the present study supports the view that work engagement influences job performance, as well as the mediating role of job crafting. The current study takes this knowledge one step further by revealing that not all types of job-crafting behaviors are equally efficient and not all types are adequate for specific working environments, such as nursing. Moreover, job-crafting behaviors could vary in their adequacy as a function of features of different working environments. As we discuss below, in the limitations section, perhaps not all of the proactive behaviors could be equally acceptable in clinical settings, due to the organizational constraints or the patients’ needs. In this vein, previous studies have shown that emotional intelligence could be considered a relevant variable in explaining work-engagement variability and should also be taken into account in applying job crafting at work [17].

### Limitations and Suggestions for Future Research and Application

First, the use of self-reported surveys is associated with common method bias and other risks of bias. Nevertheless, this study was aimed at exploring the role of nurses’ perceptions about work engagement and job performance, as well as the level of job-crafting behaviors. Hence, the use of perceptual (i.e., subjective) instruments represented the most appropriate choice to investigate those constructs. Moreover, the relatively small sample might have diminished the statistical power of the analyses and accordingly the chance to generalize the obtained results to the whole nursing population. On the other hand, it should be viewed as a first effort to investigate the role of different job-crafting dimensions with reference to their relative impact on job performance within the nursing context. Future research should include this additional facet of comparison between crafting dimensions by including all the measures at the same time as potential mediators between work engagement and job performance. Although the application of a cross-sectional design does not allow us to significantly enrich the ongoing debate over the process leading to job performance by shedding light on the sequential role played by different dimensions of job crafting, this preliminary evidence suggests the need for future longitudinal studies.

Another limitation of our study is the absence of complete information about the type of hospital (public vs. private), data that were only collected in Study 2, precluding us from obtaining any conclusions about the potential differences in job-crafting behavior among nurses working in these two types of organizations [37]. Finally, our three studies were conducted in Spain and included only Spanish-speaking nurses. On one hand, this design provides great homogeneity to our sample, but on the other hand, some cultural differences between the Spanish population and the English-speaking population, where the job-crafting theory and instruments were originally developed [38], could affect our findings [39].

One of the most relevant points under debate is still the potential conflict between nurses’ job-crafting behavior and standardized medical procedures established in clinical settings. On one hand, standardization is necessary to avoid any medical errors that could negatively affect patients’ health. On the other hand, several facets of organizational decisions and nursing practices could be adapted in order to improve both workers’ and patients’ well-being. For instance, during patients’ hospitalization, concentration of the interventions of nurses on the patient, reduction of light or noises, and patient sleep interruptions would positively impact worker fatigue and patient repose. Experienced nurses could use their acquired knowledge to better adapt their practices in these senses. This study permits a new outlook on the way to appropriately adapt job-crafting behaviors to specific job characteristics and in turn prevent the incidence of any harmful consequences on desirable outcomes as performance. As the literature on proactivity has stated, not all types of self-initiated and proactive behavior are adaptative for the employee and adequate for the organization. In the same vein, our results are promissory because they highlight the relevance of continued research on collaborative job crafting as a way to reduce the possibility of medical errors as a consequence of inadequate job-crafting practices. Hence, more research is needed in this field to design programs that promote job-crafting behavior, but specifically devoted to nurses. In the same vein, continued debate on the concept of job crafting and the comparison among different job-crafting dimensions is still necessary [40,41], specifically applied to diverse working environments, such as nursing, education, or policing, instead of empirical studies on mixed types of employees.

## 5. Conclusions

To sum up, as the JDR approach proposed, the present study supports the view that work engagement influences job performance, as well as the mediating role of job crafting. The current study takes this knowledge one step further by revealing that not all types of job-crafting behaviors are equally efficient and not all types are adequate for specific working environments, such as nursing.

## Figures and Tables

**Table 1:** Demographic characteristics of participants.

Demographic Characteristic	Study 1	Study 2	Study 3
Gender	35.5% male, 64.5% female	29.5% male, 69.5% female	35.1% male, 64.9% female
Age	Mean 41.7, SD 8.4	Mean 41.6, SD 9.6	Mean 38.7, SD 9.4
Age (categorical)	4.4 % below 26 years, 32% below 40 years, 56.4% below 55 years, 7.2% above 55 years, and 0.8% missing	5.5 % below 26 years, 34% below 40 years, 50.8% below 55 years, 9.7% above 55 years, and 0.5% missing	4.9 % below 26 years, 28.8% below 40 years, 60.5% below 55 years, 5.9% above 55 years, and 0.6% missing
Hospital tenure	Mean 12.2, SD 9.3	Mean 12.6, SD 10.3	Mean 10.6, SD 8.3.
Professional category	Practical nurses 47.3%, Registered nurses 51.2%	Practical nurses 41%, registered nurses 58%	Practical nurses 25.6%, registered nurses 74.2%
Educational level	Bachelor’s degree in nursing 7.7%, technical degree in nursing 14.1%, diploma in nursing 62.7%, master’s in nursing 15.6%	Bachelor’s degree in nursing 6.8%, technical degree in nursing 8.6%, diploma in nursing 57.2%, master’s in nursing 27.3%	Bachelor degree in nursing 10.7%, technical degree in nursing 12%, diploma in nursing 60.7%, master’s in nursing 16.6%
Type of hospital	n.a.	Private 15.1%, public 84.9%	n.a.
Region	n.a.	Madrid 13.5%, Castile 11.4%, Catalonia 6.4%, Basque Country 2%, Andalusia 63.4%	n.a.

n.a.: not available.

**Table 2:** Study 1 Pearson’s correlation matrix (*N* = 699).

	1	2	3	4	5	6	7	8
**1. Age**	1							
**2. Hospital tenure**	0.057	1						
**3. Work engagement**	0.050	−0.076	*0.92*					
**4. Job performance**	−0.002	−0.115 **	0.194 **	*0.83*				
**5. JC Increase structural resources ^1^**	0.010	−0.006	0.546 **	0.346 **	*0.82*			
**6. JC Decrease hindering demands ^1^**	−0.032	0.036	−0.171 **	−0.035	0.010	0.77		
**7. JC Increase social resources ^1^**	−0.201 **	0.017	0.329 **	0.049	0.247 **	0.058	*0.76*	
**8. JC Increase challenging demands ^1^**	−0.001	0.014	0.511 **	0.241 **	0.539 **	−0.074 *	0.324 **	0.82

^1^ JC: Job crafting. * *p* < 0.05; ** *p* < 0.001. Values in italics in the diagonal are the Cronbach’s alpha coefficients.

**Table 3:** Study 2 Pearson’s correlation matrix (*N* = 498).

	1	2	3	4	5	6
**1. Age**	1					
**2. Hospital tenure**	0.776 **	1				
**3. Work engagement**	−0.021	−0.009	0.91			
**4. Job performance**	0.028	0.083	0.401 **	0.82		
**5. JC individual ^1^**	−0.019	−0.022	0.421 **	0.279 **	0.78	
**6. JC collaborative ^1^**	0.061	0.095 *	0.395 **	0.230 **	0.571 **	0.89

^1^ JC: Job crafting. * *p* < 0.05; ** *p* < 0.001. Values in italics in the diagonal are the Cronbach’s apha coefficients.

**Table 4:** Study 3 Pearson’s correlation matrix (*N* = 308).

	1	2	3	4	5	6	7
**1. Age**	1						
**2. Hospital tenure**	0.659 **	1					
**3. Work engagement**	0.044	−0.004	*0.89*				
**4. Job performance**	−0.009	−0.006	0.184 **	*0.84*			
**5. JC cognitive** ^1^	0.016	0.009	0.617 **	0.158 **	*0.86*		
**6. JC task-related ^1^**	0.016	0.002	0.563 **	0.235 **	0.539 **	*0.82*	
**7. JC relational ^1^**	0.051	0.082	0.459 **	0.190 **	0.513 **	0.460 **	*0.85*

^1^ JC: Job crafting. ** *p* < 0.001. Values in italics in the diagonal are the Cronbach’s alpha coefficients.

## Data Availability

Not applicable.

## References

[1] Rodríguez-Pérez M., Mena-Navarro F., Domínguez-Pichardo A., Teresa-Morales C. (2022). Current Social Perception of and Value Attached to Nursing Professionals’ Competences: An Integrative Review. Int. J. Environ. Res. Public Health.

[2] Huerta-González S., Selva-Medrano D., López-Espuela F., Caro-Alonso P.Á., Novo A., Rodríguez-Martín B. (2021). The psychological impact of Covid-19 on front line nurses: A synthesis of qualitative evidence. Int. J. Environ. Res. Public Health.

[3] Ramírez-Elvira S., Romero-Béjar J.L., Suleiman-Martos N., Gómez-Urquiza J.L., Monsalve-Reyes C., Cañadas-De la Fuente G.A., Albendín-García L. (2021). Prevalence, risk factors and burnout levels in intensive care unit nurses: A systematic review and meta-analysis. Int. J. Environ. Res. Public Health.

[4] Ghazzawi R., Bender M., Daouk-Öyry L., van de Vijver F.J., Chasiotis A. (2021). Job crafting mediates the relation between creativity, personality, job autonomy and well-being in Lebanese nurses. J. Nurs. Manag..

[5] Munir M.M., Rubaca U., Munir M.H. (2021). A Systematic Review of Job Crafting, Job Characteristics, Work Engagement, and Exhaustion of Female Nurses during COVID-19 Pandemic. J. Educ. Res. Soc. Sci. Rev. (JERSSR).

[6] Harbridge R., Ivanitskaya L., Spreitzer G., Boscart V. (2022). Job crafting in registered nurses working in public health: A qualitative study. Appl. Nurs. Res..

[7] Tims M., Bakker A.B. (2010). Job crafting: Towards a new model of individual job redesign. SA J. Ind. Psychol..

[8] Tims M., Bakker A.B., Derks D. (2012). Development and validation of the job crafting scale. J. Vocat. Behav..

[9] Leana C., Appelbaum E., Shevchuk I. (2009). Work process and quality of care in early childhood education: The role of job crafting. Acad. Manag. J..

[10] Alonso C., Fernández-Salinero S., Topa G. (2019). The impact of both individual and collaborative job crafting on Spanish teachers’ well-being. Educ. Sci..

[11] Wrzesniewski A., Dutton J.E. (2001). Crafting a job: Revisioning employees as active crafters of their work. Acad. Manag. Rev..

[12] Slemp G.R., Vella-Brodrick D.A. (2013). The Job Crafting Questionnaire: A new scale to measure the extent to which employees engage in job crafting. Int. J. Wellbeing.

[13] Bakker Arnold B., Sanz-Vergel A. (2013). Weekly work engagement and flourishing: The role of hindrance and challenge job demands. J. Vocat. Behav..

[14] Mazzetti G., Robledo E., Vignoli M., Topa G., Guglielmi D., Schaufeli W.B. (2021). Work engagement: A meta-analysis using the job demands-resources model. Psychol. Rep..

[15] García-Sierra R., Fernández-Castro J., Martínez-Zaragoza F. (2016). Work engagement in nursing: An integrative review of the literature. J. Nurs. Manag..

[16] Gómez-Salgado J., Navarro-Abal Y., López-López M.J., Romero-Martín M., Climent-Rodríguez J.A. (2019). Engagement, passion and meaning of work as modulating variables in nursing: A theoretical analysis. Int. J. Environ. Res. Public Health.

[17] Pérez-Fuentes M.D.C., Molero Jurado M.D.M., Gázquez Linares J.J., Oropesa Ruiz N.F. (2018). The role of emotional intelligence in engagement in nurses. Int. J. Environ. Res. Public Health.

[18] Martos Martínez Á., Pérez-Fuentes M.D.C., Molero Jurado M.D.M., Simón Márquez M.D.M., Barragán Martín A.B., Gázquez Linares J.J. (2021). Empathy, affect and personality as predictors of engagement in nursing professionals. Int. J. Environ. Res. Public Health.

[19] Wu W.L., Lee Y.C. (2020). How spiritual leadership boosts nurses’ work engagement: The mediating roles of calling and psychological capital. Int. J. Environ. Res. Public Health.

[20] Zeng D., Takada N., Hara Y., Sugiyama S., Ito Y., Nihei Y., Asakura K. (2022). Impact of Intrinsic and Extrinsic Motivation on Work Engagement: A Cross-Sectional Study of Nurses Working in Long-Term Care Facilities. Int. J. Environ. Res. Public Health.

[21] Bernburg M., Hetzmann M.S., Mojtahedzadeh N., Neumann F.A., Augustin M., Harth V., Groneberg D.A., Zyriax B.-C., Mache S. (2021). Stress perception, sleep quality and work engagement of German outpatient nurses during the COVID-19 pandemic. Int. J. Environ. Res. Public Health.

[22] Tims M., Bakker A.B., Derks D. (2013). The impact of job crafting on job demands, job resources, and well-being. J. Occup. Health Psychol..

[23] Rudolph C.W., Katz I.M., Lavigne K.N., Zacher H. (2017). Job crafting: A meta-analysis of relationships with individual differences, job characteristics, and work outcomes. J. Vocat. Behav..

[24] Cheng J.C., Chen C.Y., Teng H.Y., Yen C.H. (2016). Tour leaders’ job crafting and job outcomes: The moderating role of perceived organizational support. Tour. Manag. Perspect..

[25] Mäkikangas A., Bakker A.B., Schaufeli W.B. (2017). Antecedents of daily team job crafting. Eur. J. Work. Organ. Psychol..

[26] Letona-Ibañez O., Carrasco M., Martinez-Rodriguez S., Amillano A., Ortiz-Marques N. (2019). Cognitive, relational and task crafting: Spanish adaptation and analysis of psychometric properties of the Job Crafting Questionnaire. PLoS ONE.

[27] Chen C.Y., Yen C.H., Tsai F.C. (2014). Job crafting and job engagement: The mediating role of person-job fit. Int. J. Hosp. Manag..

[28] Llorente-Alonso M., Gabriela T. (2019). Individual crafting, collaborative crafting, and job satisfaction: The mediator role of engagement. J. Work. Organ. Psychol..

[29] Schachler V., Epple S.D., Clauss E., Hoppe A., Slemp G.R., Ziegler M. (2019). Measuring job crafting across cultures: Lessons learned from comparing a German and an Australian sample. Front. Psychol..

[30] Slemp G.R., Vella-Brodrick D.A. (2014). Optimising employee mental health: The relationship between intrinsic need satisfaction, job crafting, and employee well-being. J. Happiness Stud..

[31] Slemp G.R., Kern M.L., Vella-Brodrick D.A. (2015). Workplace well-being: The role of job crafting and autonomy support. Psychol. Well-Being.

[32] Schaufeli W.B., Bakker A.B. (2003). Utrecht work engagement scale: Preliminary manual. Occupational Health Psychology Unit 2003. Utrecht Univ. Utrecht.

[33] Williams L.J., Anderson S.E. (1991). Job satisfaction and organizational commitment as predictors of organizational citizenship and in-role behaviors. J. Manag..

[34] Demerouti E., Rispens S. (2014). Improving the image of student-recruited samples: A commentary. J. Occup. Organ. Psychol..

[35] Hayes A.F. (2013). The PROCESS macro for SPSS and SAS (version 2.13) [Software].

[36] Zhang F., Parker S.K. (2019). Reorienting job crafting research: A hierarchical structure of job crafting concepts and integrative review. J. Organ. Behav..

[37] Esteves T., Pereira Lopes M. (2017). Leading to crafting: The relation between leadership perception and nurses’ job crafting. West. J. Nurs. Res..

[38] Beaton D.E., Bombardier C., Guillemin F., Ferraz M.B. (2000). Guidelines for the process of cross-cultural adaptation of self-report measures. Spine.

[39] Sakuraya A., Shimazu A., Eguchi H., Kamiyama K., Hara Y., Namba K., Kawakami N. (2017). Job crafting, work engagement, and psychological distress among Japanese employees: A cross-sectional study. BioPsychoSoc. Med..

[40] Bruning P.F., Campion M.A. (2018). A role–resource approach–avoidance model of job crafting: A multimethod integration and extension of job crafting theory. Acad. Manag. J..

[41] Lichtenthaler P.W., Fischbach A. (2019). A meta-analysis on promotion-and prevention-focused job crafting. Eur. J. Work. Organ. Psychol..

